# A frequency restoration control scheme of series-parallel-type microgrids with local low bandwidth communication

**DOI:** 10.1038/s41598-026-38888-8

**Published:** 2026-02-06

**Authors:** Lang Li, Shixun Shen, Peng Tian, Ke Zhou

**Affiliations:** School of Automation Engineering, Moutai Institute, Renhuai, 564507 China

**Keywords:** Electrical and electronic engineering, Energy grids and networks

## Abstract

Series-parallel-type microgrids include several distributed generators (DGs) connected in series to form a string, with multiple such strings then connected in parallel. The existing centralized frequency restoration control needs global communications. To reduce the communication requirements of frequency restoration control for series-parallel-type microgrids, this paper proposes a frequency restoration control scheme that relies solely on local low bandwidth communication (LBC). Specifically, the first DG in each string utilizes the local LBC network to achieve consensus control, while the remaining DGs operate without any communications. In a series-connected system, the line current serves as a common information. Global frequency restoration can be achieved by enabling only the first DG’s line current in each string to perform frequency recovery control. Compared to existing global communication-based methods, the proposed method ensures frequency restoration using only the local LBC for the first DG in each string, significantly reducing the need for communication. Meanwhile, the system’s computational complexity is reduced. Further, the stability of the proposed controller is validated based on the root locus method. Finally, simulation results with the proposed method validated the frequency restoration performance, applicability to RL and RC loads, robustness under certain communication failures, and proportional active power allocations.

## Introduction

In recent years, renewable energy has experienced rapid development, particularly in photovoltaics (PVs) and wind turbines^1–3^. However, renewable energy is characterized by randomness and volatility^4^. To achieve stable waveforms of electrical power, power electronic converters have become essential^5^. Microgrids, which integrate generation, transmission, distribution, utilization, and storage, can effectively accommodate various renewable energy sources^6,7^.

With high penetration of inverter-based resources (IBRs), microgrid’s stability becomes more challenging due to their low-inertia characteristics^8^. In particular, stability issues may arise from weak-grid conditions, impedance interactions, operating-mode transitions and disturbances^9^. Accordingly, recent studies often assess IBR-dominated microgrids from both the frequency domain and the time domain^9^. IBRs can be broadly categorized into grid-following inverters (GFLIs) and grid-forming inverters (GFMIs)^10^. GFLIs typically rely on synchronization mechanisms such as PLL-based schemes, which may suffer from degraded stability margins in weak grids^11^. In contrast, GFMIs directly establish voltage and frequency references^12^, which are commonly controlled using droop control, VSG-based schemes, VOC, and so on^13^.

Beyond primary control, secondary frequency and voltage restoration is essential to eliminate steady-state deviations introduced by droop characteristics^14,15^. Depending on the communication, restoration strategies can be categorized as centralized, distributed, and decentralized^16^. The reliance on communication networks renders centralized control susceptible to single-point communication failures. Conversely, distributed strategies, requiring only local communication, and decentralized strategies, which eliminate communication requirements, effectively mitigate the impact of communication dependence on system stability^17^. To clarify their trade-offs and typical applications, centralized and non-centralized strategies for frequency and voltage restoration are summarized and compared in Table 1.

Paralleled-type microgrid consists of multiple inverters connected in parallel to deliver power to the load^18,19^. Droop control, as a typical communication-free control method, can achieve frequency self-synchronization of the system. In inductive distribution lines, P-f droop control is required^20^. In resistive lines, Q-f droop control is applied^21^. When the system load increases, the frequency decreases with droop controllers, causing the system frequency to deviate from its reference value. Communication-based methods are necessary to compensate for the frequency deviations. A distributed event-triggered control is proposed to obtain economic operation and frequency restoration^22^, which requires communication among neighboring agents. To tackle both economic dispatch and frequency restoration, a model predictive control is proposed^23^. A local frequency restoration strategy based on an event-driven method is presented^24^. Several distributed secondary control schemes are proposed^25–27^, addressing delay tolerance, optimal load dispatch, and finite-time robustness. For the paralleled-type microgrids, communications between all distributed generators (DGs) are necessary to achieve frequency restoration.

The cascaded-type microgrids consist of multiple low-voltage DGs in series connection^28,29^. Droop control is introduced to achieve frequency synchronization^30^. Additionally, a power factor angle consistency droop control is presented^31^, which can maintain a constant load voltage amplitude. However, as the load changes, the frequency may diverge from the reference value. Further, a communication-free economic dispatch control method is proposed^32^, which can realize the frequency restoration and load voltage quality. From the above, it can be concluded that the cascaded-type microgrids can achieve frequency restoration using a communication-free approach.

**Table 1:** Comparison of centralized, distributed, decentralized frequency and voltage control strategies.

Aspect	Centralized restoration	Distributed restoration	Decentralized control
Control objective	Eliminate steady-state frequency/voltage deviations caused by primary droop control	Eliminate steady-state frequency/voltage deviations caused by primary droop control	Frequency synchronization/ Construction voltage waveform
Communication requirement	Global communication with all DGs	Adjacent communication among neighboring DGs	No communication required for DGs
Advantages	High restoration accuracy; Fast recovery; strong coordination	Improved robustness; good scalability; reduced communication burden	High reliability; simple implementation; communication-free
Limitations	Vulnerable to single-point failures; high communication dependency	Frequency/voltage restoration depends on communication topology; convergence may be slower	Voltage/frequency deviation
Typical applications	Microgrids with hierarchical control and limited number of nodes	Medium- to large-scale microgrids	Communication-constrained or islanded microgrids

**Fig. 1:**
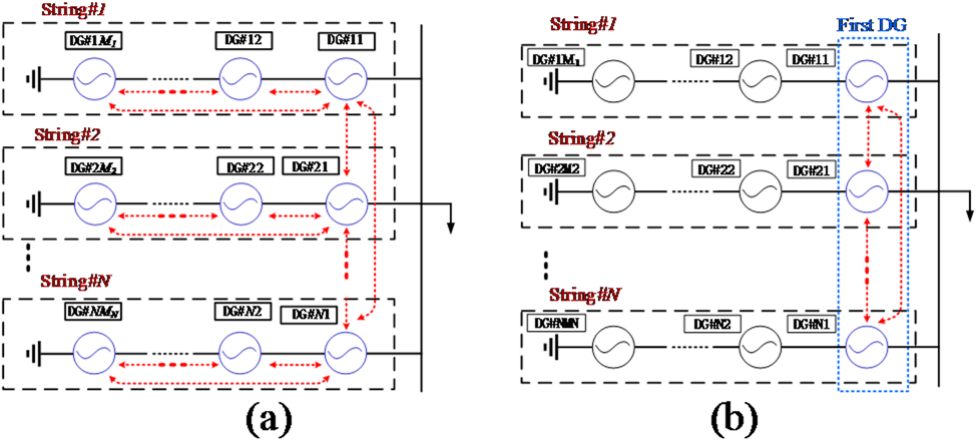
Graphical diagram of communication network. **(a)** existing method^37^ **(b)** proposed control method.

Series-parallel-type microgrids comprise multiple DGs connected in series to form a string-generation unit, with several such string-generation units connected in parallel^33^. A hierarchical power-sharing control method is presented^34^, in which the upper controller regulates the parallel string converters and the lower controller shares the load power among DGs in series connection. A locally distributed control is proposed^35^, where the string DG requires the neighboring communication to exchange data, and string generation units are regulated without communication. A power-sharing and frequency synchronization control for series-parallel-type microgrids is presented^36^. However, these methods would result in frequency deviation^34–36^ and fail to provide frequency restoration capability. To realize frequency restoration, a unified distributed control for a series-parallel-type microgrid is proposed^37^ with RL and RC loads (See Fig. 1(a)). In^37^, globally distributed communication networks are needed for all DGs in series-parallel-type microgrids to realize frequency restoration. As seen above, the limitation of existing frequency restoration control for series-parallel-type microgrids lies in their reliance on a globally distributed communication network among DGs. When communication failures occur, the system may become unstable, and the computational complexity of the system is relatively high.

To address the above limitation, it is essential to explore a new frequency restoration control scheme to further reduce the need for communication. The main contributions of this paper is the proposal of a frequency restoration control, where only local low-bandwidth communication (LBC) is required (See Fig. 1(b)). The LBC is employed to exchange only essential information among neighboring units, which significantly reduces communication requirements while maintaining effective coordination. The local LBC network is required for the first DG in each string to achieve consensus regulation. The rest DGs are controlled in a communication-free approach. Therefore, the frequency restoration is obtained by only using the local LBC for the first DG in each string. Compared to^36^, the proposed method in this paper holds the frequency restoration capability. Compared to the existing global communication-based methods^37^, the proposed method requires significantly less communication. For each string in series-parallel-type microgrids, the existing control method^37^ requires every DG to communicate, whereas the proposed control method only requires the first DG to communicate. Thus, this approach achieves a notable reduction in both communication overhead and computational complexity, which enhances reliability and reduces the initial investment costs of the system.

The paper is structured: Sect. 2 presents the configuration of the series-parallel-type microgrid. Section 3 presents the proposed frequency restoration control scheme with local low-bandwidth communication. Section 4 conducts the Stability analysis. Simulations are presented in Sect. 5, while Sect. 6 provides the conclusion.

## Configuration of series-parallel-type microgrid

**Fig. 2:**
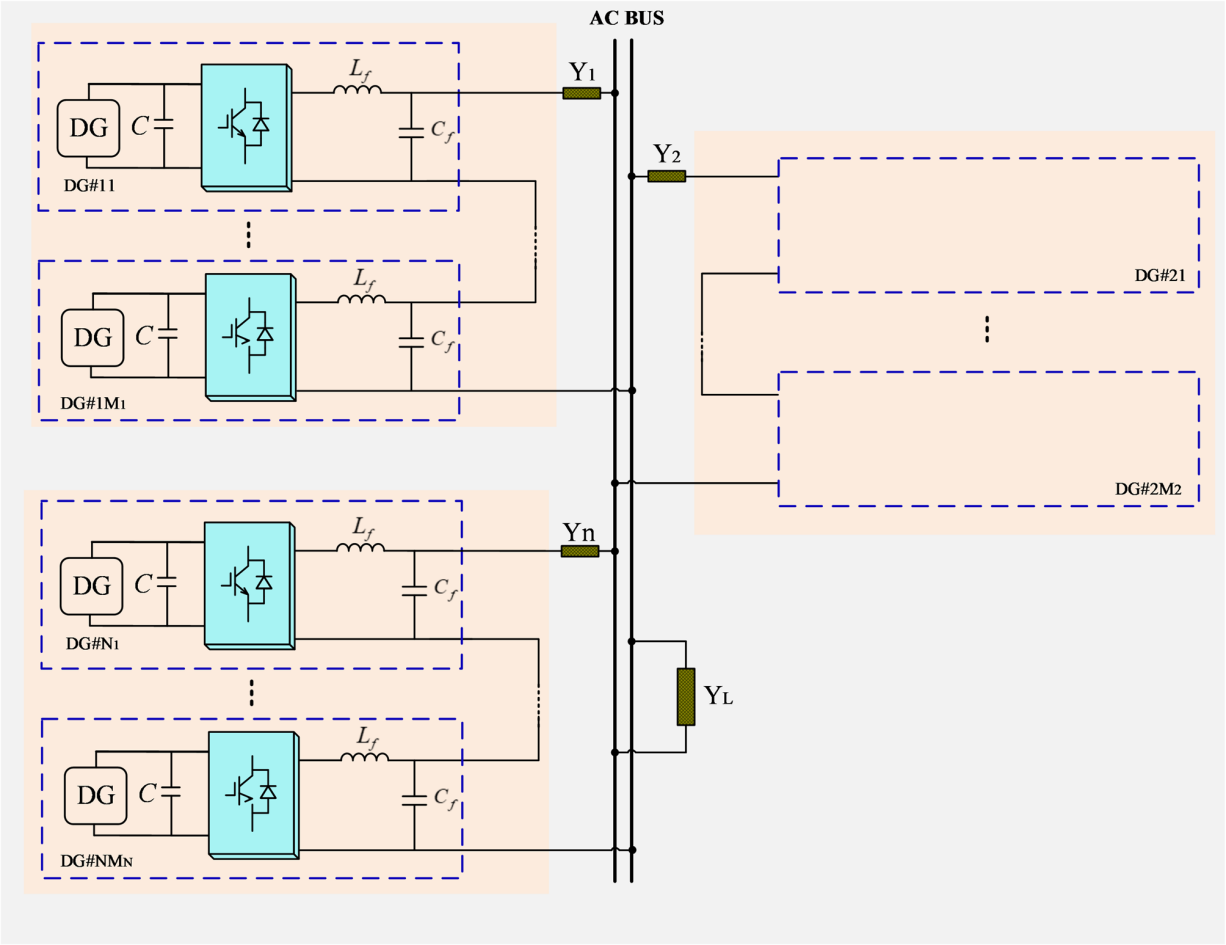
Configuration of an islanded series-parallel-type microgrid.

The structure diagram of an islanded series-parallel-type microgrid is illustrated in Fig. 1, in which there are *N* strings, and each string includes *M* DGs. Each DG unit consists of an H-bridge converter and an LC filter, and is connected to the AC bus via a line to supply power to the common loads. In each string of power generation units, only the first DG communicates and interacts with the first DGs of other strings, adopting a ring-type distributed communication architecture. From Fig. 2, the power of DG*#ij* is^33^:

1 $${P_{ij}}+j{Q_{ij}}={V_{ij}}{e^{j{\delta _{ij}}}}{\left[ {\left( {\sum\limits_{{b=1}}^{M} {{V_{ib}}{e^{j{\delta _{ib}}}} - } {V_p}{e^{j{\delta _p}}}} \right)\left| {{Y_i}} \right|{e^{j{\varphi _i}}}} \right]^*}$$

where${P_{ij}}$indicates active powers,${Q_{ij}}$means reactive powers,${V_p}{e^{j{\delta _p}}}$is defined as the voltage at PCC. $\left| {{Y_i}} \right|{e^{j{\varphi _i}}}$refers to equivalent line admittances. In this paper, the line impedances are assumed to be inductive, ${\varphi _i} \approx - {\pi \mathord{\left/ {\vphantom {\pi 2}} \right. \kern-0pt} 2}$.

2 $${V_p}{e^{j{\delta _p}}}=\sum\limits_{{a=1}}^{N} {\sum\limits_{{b=1}}^{M} {\left( {\frac{{{Y_a}{V_{ab}}{e^{j{\delta _{ab}}}}}}{{{Y_{load}}+\sum\limits_{{c=1}}^{N} {{Y_c}} }}} \right)} }$$

where

3 $$\frac{{{Y_a}}}{{{Y_{load}}+\sum\limits_{{c=1}}^{N} {{Y_c}} }}=\left| {{{Y^{\prime}}_a}} \right|{e^{j{{\varphi ^{\prime}}_a}}}$$

In (3),${Y_i}$ and ${Y_{load}}$ represents the admittance of transmission line and loads.

Equation (2) can be rewritten as:

4 $${V_p}{e^{j{\delta _p}}}=\sum\limits_{{a=1}}^{N} {\sum\limits_{{b=1}}^{M} {\left| {{{Y^{\prime}}_a}} \right|{V_{ab}}{e^{j\left( {{\delta _{ab}}+{{\varphi ^{\prime}}_a}} \right)}}} }$$

The power transmission characteristic can be denoted as,

5 $$\begin{gathered} {p_{ij}}=\left| {{Y_i}} \right|{V_{ij}}\sum\limits_{{a=1}}^{N} {\sum\limits_{{b=1}}^{M} {\left| {{{Y^{\prime}}_a}} \right|{V_{ab}}\sin \left( {{\delta _{ij}} - {\delta _{ab}} - {{\varphi ^{\prime}}_a}} \right)} } \hfill \\ {\kern 1pt} {\kern 1pt} {\kern 1pt} {\kern 1pt} {\kern 1pt} {\kern 1pt} {\kern 1pt} {\kern 1pt} {\kern 1pt} {\kern 1pt} {\kern 1pt} {\kern 1pt} {\kern 1pt} {\kern 1pt} {\kern 1pt} {\kern 1pt} {\kern 1pt} {\kern 1pt} {\kern 1pt} {\kern 1pt} - \left| {{Y_i}} \right|{V_{ij}}\sum\limits_{{c=1}}^{M} {{V_{ic}}\sin \left( {{\delta _{ij}} - {\delta _{ic}}} \right)} \hfill \\ \end{gathered}$$

6 $$\begin{gathered} {q_{ij}}= - \left| {{Y_i}} \right|{V_{ij}}\sum\limits_{{a=1}}^{N} {\sum\limits_{{b=1}}^{M} {\left| {{{Y^{\prime}}_a}} \right|{V_{ab}}\cos \left( {{\delta _{ij}} - {\delta _{ab}} - {{\varphi ^{\prime}}_a}} \right)} } \hfill \\ {\kern 1pt} {\kern 1pt} {\kern 1pt} {\kern 1pt} {\kern 1pt} {\kern 1pt} {\kern 1pt} {\kern 1pt} {\kern 1pt} {\kern 1pt} {\kern 1pt} {\kern 1pt} {\kern 1pt} {\kern 1pt} {\kern 1pt} {\kern 1pt} {\kern 1pt} {\kern 1pt} {\kern 1pt} {\kern 1pt} +\left| {{Y_i}} \right|{V_{ij}}\sum\limits_{{c=1}}^{M} {{V_{ic}}\cos \left( {{\delta _{ij}} - {\delta _{ic}}} \right)} \hfill \\ \end{gathered}$$

Let${\omega _c}$as a cutoff frequency. Then, filtered active powers ${P_{ij}}$ and reactive powers ${Q_{ij}}$ are^32^:

7 $${P_{ij}}={p_{ij}}\frac{{{\omega _c}}}{{s+{\omega _c}}}$$

8 $${Q_{ij}}={q_{ij}}\frac{{{\omega _c}}}{{s+{\omega _c}}}$$

In order to restore the frequency of series-parallel-type microgrids, the problem formulation is expressed as:

9 $$\mathop {\lim }\limits_{{t \to \infty }} \left( {{\omega ^*} - {\omega _{ij}}} \right)=0$$

where${\omega _{ij}}$represents the angular frequency of the DG#*ij*, ${\omega ^*}$ is the reference frequency.

## Proposed frequency restoration control with local low-bandwidth communication

To solve the frequency restoration problem (9) of series-parallel-type microgrids, the proposed strategy is offered in this Section. For the first DG in each string, a distributed control based on low-bandwidth communication is adopted. For the rest DGs in each string, a decentralized control is used.

For simplicity, the proposed method is developed under the following assumptions: (1) ${Q_{ij}} \ne 0$; (2) the inner-loops controller ensures zero-error tracking of each DG’s output voltage to its reference; (3) the phase-locked loop (PLL) achieves fast and zero-error locking of the angular frequency of the local line current.

### The proposed frequency restoration control with local low-bandwidth communication

**Fig. 3:**
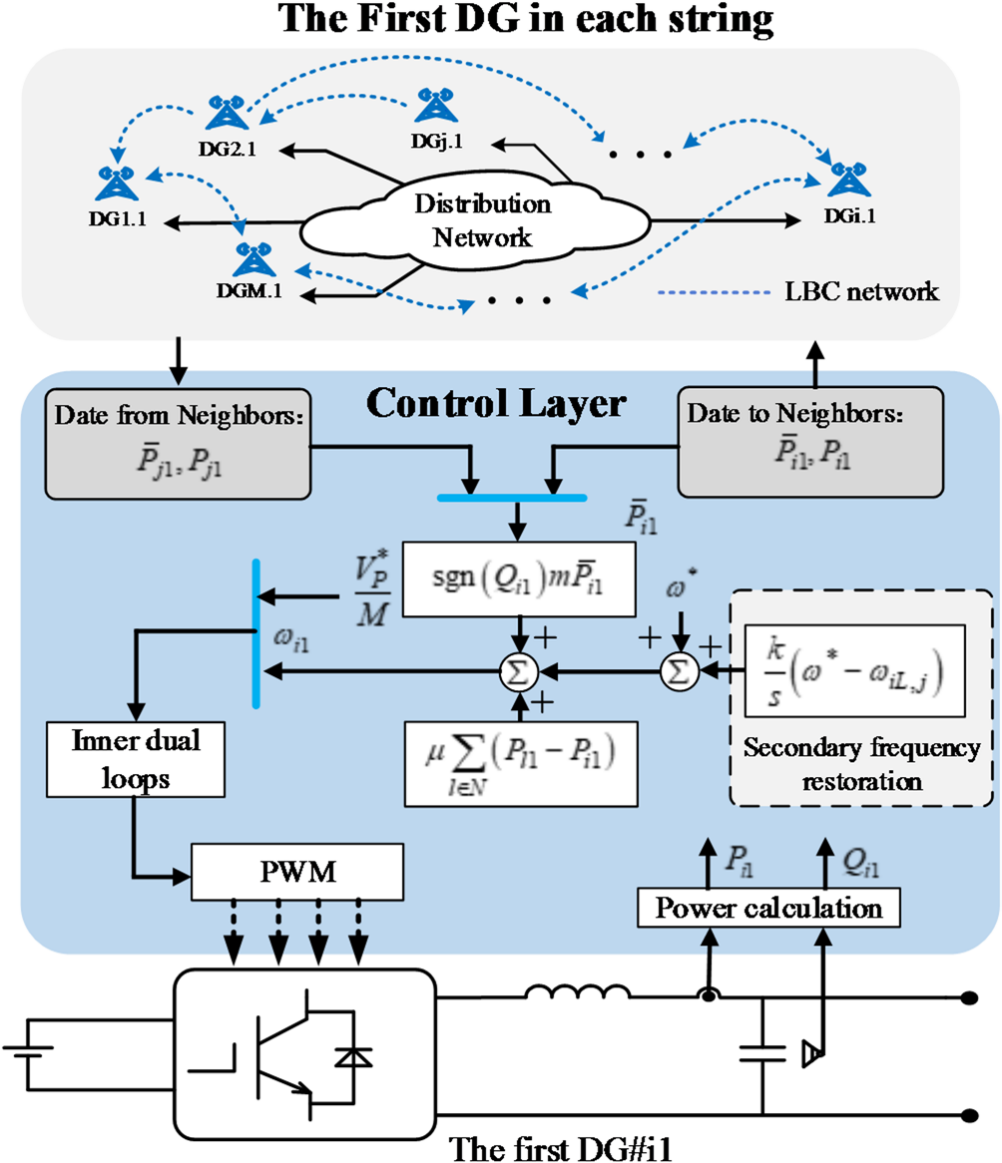
Control diagram of the DG#i1.

For the first DG#*ij* (*j =* 1) in each string, the proposed frequency restoration control method is expressed as:

10 $${\omega _{ij}}={\omega ^*}+\operatorname{sgn} \left( {{Q_{ij}}} \right)m{\bar {P}_{ij}}+\mu \sum\limits_{{l \in N}} {\left( {{P_{l1}} - {P_{ij}}} \right)+\frac{k}{s}\left( {{\omega ^*} - {\omega _{iL,j}}} \right)}$$

11 $${V_{ij}}=\frac{{V_{P}^{*}}}{M}$$

where$V_{P}^{*}$ is reference load voltage. *m*, $\mu$, *k* are the positive constants.$\operatorname{sgn} \left( \cdot \right)$is a sign function.${\omega _{iL,j}}$is the local measurement frequency of the transmission line current. ${\bar {P}_{ij}}$ denotes the mean observed active power for the first DG across different strings, which can be written as^37^:

12 $${\bar {P}_{i1}}={P_{i1}}+\int\limits_{0}^{t} {\sum\limits_{{b \in N}} {{a_{ij}}} \left( {{{\bar {P}}_{b1}}\left( \tau \right) - {{\bar {P}}_{i1}}\left( \tau \right)} \right)} d\tau$$

where${a_{ij}}$represents communication weights. Only the first DG of each string utilizes the LBC to manage date variations, and its control diagram is illustrated in Fig. 3.

For the rest DG#*ij* in each string ($j=2,3, \cdots ,M$), the proposed method is written as:

13 $$\left\{ \begin{gathered} {\omega _{ij}}={\omega ^*}+\operatorname{sgn} \left( {{Q_{ij}}} \right)m{P_{ij}}+\frac{k}{s}\left( {{\omega ^*} - {\omega _{iL,j}}} \right) \hfill \\ {V_{ij}}=\frac{{V_{P}^{*}}}{M} \hfill \\ \end{gathered} \right.$$

**Fig. 4:**
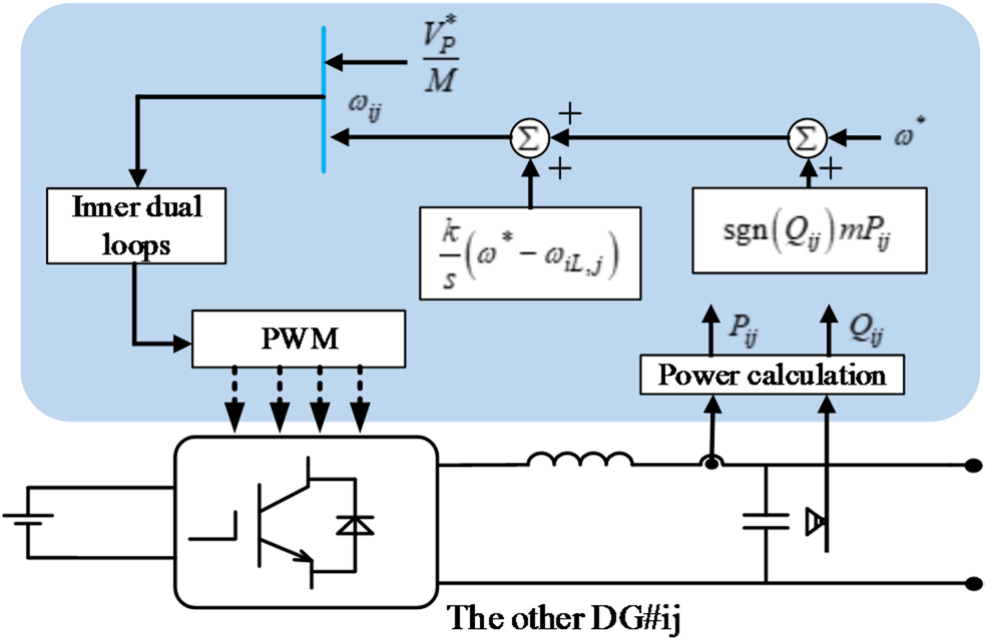
Control schematic of the DG#ij.

From (13), all subscripts are related to *ij*, the construction of the proposed controller only needs the local information. As a result, all DGs except for the first are controlled in a communication-free manner. The control diagram for the other DGs in the *i*^*th*^ string is shown in Fig. 4.

According to (10) and (13), only the first DG requires LBC to share information with others. The other DGs are regulated without the need for communication. Therefore, the introduced scheme holds improved reliability compared to the global distributed control.

Based on the common current in each string, the frequency restoration is obtained in a decentralized manner. Meanwhile, the low bandwidth communication for the first DG in each string is applied to realize the global frequency restoration. The existing method needs distributed communication links for all DGs in each string^37^. In the proposed scheme, only the first DG in each string requires communication from the neighboring first DG. The rest DGs in each string are a fully communication-free approach. Therefore, the communication requirements and computational complexity of the controller are reduced. Compared to the existing method, the proposed method enhances system reliability and reduces investment costs.

**Fig. 5:**
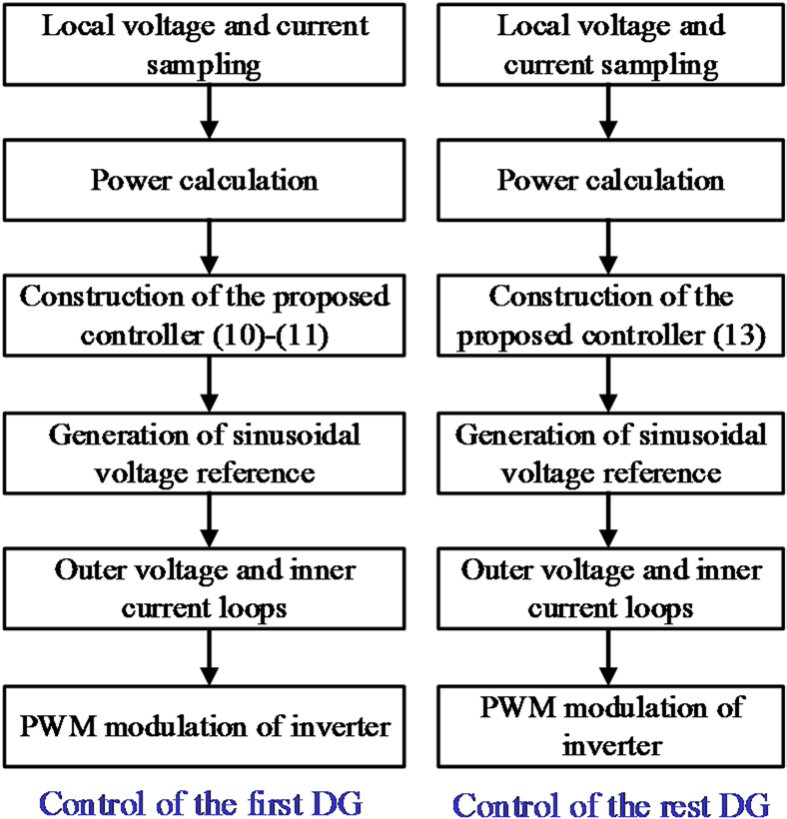
Flowchart of the proposed control scheme.

The overall control flowchart of the proposed method is illustrated in Fig. 5. The local power is first calculated based on the measured voltage and current, and then proposed controller is constructed according to the power calculation and neighboring information.

### Steady-state analysis

From (12), when the microgrid reaches a steady state, there is

14 $${\bar {P}_{11}}={\bar {P}_{21}}= \cdots ={\bar {P}_{N1}}=\frac{{{P_{11}}+{P_{21}}+ \cdots +{P_{N1}}}}{N}$$

As reported in^35^, the observed value will converge to its actual value in the steady state:

15 $${P_{i1}}={P_{j1}}={\bar {P}_{i1}}={\bar {P}_{j1}}=\frac{{{P_{11}}+{P_{21}}+ \cdots +{P_{N1}}}}{N}$$

As the microgrid transitions to the steady state, from (10) and (13), for the *i*^*th*^ and *j*^*th*^ string, there is:

16 $${\omega _{iL,1}}={\omega _{iL,2}}= \cdots ={\omega _{iL,M}}={\omega ^*}$$

17 $${\omega _{jL,1}}={\omega _{jL,2}}= \cdots ={\omega _{jL,M}}={\omega ^*}$$

Combining (16)-(17), for the DG*#ij* and DG*#ab*, there is:

18 $${\omega _{ij}}={\omega _{ab}}={\omega ^*}$$

Therefore, frequency synchronization and frequency restoration have been obtained with the proposed control method. Similar to the traditional parallel microgrid with traditional droop control^38^, the reactive power can not be shared accurately for the parallel-connected strings in the series-parallel-type microgrids due to the impedance mismatch.

In order to realize proportional active power allocations, the voltage reference in (11) is modified as:

19 $${V_{ij}}=\frac{{{M_{ij}}}}{{\sum\limits_{{j=1}}^{M} {{M_{ij}}} }}V_{P}^{*}$$

where ${M_{ij}}$is a power allocation coefficient. From ${P_{ij}}={V_{ij}}{I_{ij}}\cos {\varphi _{ij}}$, due to the series connection, the active power allocation is related to the voltage amplitudes.

## Stability analysis

To illustrate the stability of the proposed method, a root locus analysis is conducted using a small-signal model in this section.

### Model

Equation (12) can be expressed:

20 $${{\mathbf{\bar {P}}}_{\mathbf{l}}}=s{(s{{\mathbf{I}}_{\mathbf{l}}}+{{\mathbf{L}}_{\mathbf{l}}})^{ - 1}}{{\mathbf{P}}_{\mathbf{l}}}={{\mathbf{H}}_{\mathbf{l}}}{{\mathbf{P}}_{\mathbf{l}}}$$

where${{\mathbf{\bar {P}}}_{\mathbf{l}}}={[{\bar {P}_{11}}, \cdots ,{\bar {P}_{N1}}]^T}$,${{\mathbf{P}}_{\mathbf{l}}}={[{P_{11}}, \cdots ,{P_{N1}}]^T}$.${{\mathbf{I}}_{\mathbf{l}}}$is a matrix with 1 on the main diagonal and 0 elsewhere. ${{\mathbf{H}}_{\mathbf{l}}}$ means an estimator transfer function. ${{\mathbf{L}}_{\mathbf{l}}}$is a Laplacian matrix. ${{\mathbf{L}}_{\mathbf{l}}}={\mathbf{D}}_{{\mathbf{l}}}^{{{\mathbf{in}}}} - {{\mathbf{C}}_{\mathbf{l}}}$, ${\mathbf{D}}_{{\mathbf{l}}}^{{{\mathbf{in}}}}$represents an in-degree matrix. ${{\mathbf{C}}_{\mathbf{l}}}$means an adjacency matrix.

The frequency of the transmission line current can be written as^32^:

21 $${\omega _{iL,j}}=\frac{1}{M}\sum\limits_{{j=1}}^{M} {{\omega _{ij}}}$$

Assume that${\delta _s}$means the synchronized phase angle at a steady state^39]– [40^, ${\tilde {\delta }_{ij}}={\delta _{ij}} - {\delta _s}$.

For the first DG in each string, (10) can be rewritten as:

22 $${\dot {\omega }_{i1}}=\operatorname{sgn} \left( {{Q_{i1}}} \right)m{\dot {\bar {P}}_{i1}}+\mu \sum\limits_{{l \in N}} {\left( {{{\dot {P}}_{l1}} - {{\dot {P}}_{i1}}} \right)+k\left( {{\omega ^*} - \frac{1}{M}\sum\limits_{{j=1}}^{M} {{\omega _{ij}}} } \right)}$$

23 $$\Delta {\dot {\tilde {\delta }}_{i1}}=\Delta {\omega _{i1}}$$

Small signal representation of (22) is expressed as:

24 $${\mathbf{\Delta }}{{\mathbf{\dot {\omega }}}_{\mathbf{l}}}=m{{\mathbf{D}}_{\mathbf{l}}}{{\mathbf{H}}_{\mathbf{l}}}{\mathbf{\Delta }}{{\mathbf{\dot {P}}}_{\mathbf{l}}}+\mu {{\mathbf{L}}_{\mathbf{k}}}{\mathbf{\Delta }}{{\mathbf{\dot {P}}}_{\mathbf{l}}} - \frac{k}{M}{\mathbf{\Delta }}{{\mathbf{\omega }}_{{\mathbf{ia}}}}$$

where $${\mathbf{\Delta }}{{\mathbf{\dot {\omega }}}_{\mathbf{l}}}={[\Delta {\dot {\omega }_{11}},\Delta {\dot {\omega }_{21}}, \cdots ,\Delta {\dot {\omega }_{N1}}]^T}$$, $${{\mathbf{D}}_{\mathbf{l}}}=diag[\operatorname{sgn} \left( {{Q_{i1}}} \right)]$$, $${\mathbf{\Delta }}{{\mathbf{\dot {P}}}_{\mathbf{l}}}={[\Delta {\dot {P}_{11}},\Delta {\dot {P}_{21}}, \cdots ,\Delta {\dot {P}_{N1}}]^T}$$, $${\mathbf{\Delta }}{{\mathbf{\omega }}_{{\mathbf{ia}}}}=[\Delta {\omega _{i1}},\Delta {\omega _{i2}}, \cdots ,\Delta {\omega _{iM}}]$$, and,

25 $${{\mathbf{L}}_{\mathbf{k}}}=\left[ {\begin{array}{*{20}{c}} { - \left( {M - 1} \right)}& \cdots &1 \\ \vdots & \ddots & \vdots \\ 1& \cdots &{ - \left( {M - 1} \right)} \end{array}} \right]$$

The small signal model of (7) is formulated as:

26 $$\Delta {\dot {P}_{ij}}= - {\omega _c}\Delta {P_{ij}}+{\omega _c}\Delta {p_{ij}}$$

where

27 $$\Delta {p_{ij}}=\sum\limits_{{a=1}}^{N} {\sum\limits_{{b=1}}^{M} {k_{{p\_{\delta _{ab}}}}^{{ij}}\Delta {{\tilde {\delta }}_{ab}}} }$$

28 $$k_{{p\_{\delta _{ab}}}}^{{ij}}=\frac{{\partial {p_{ij}}}}{{\partial {{\tilde {\delta }}_{ab}}}},\left( \begin{gathered} i=1, \cdots ,N;j=1, \cdots ,{M_i} \hfill \\ a=1, \cdots ,N;b=1, \cdots ,{M_a} \hfill \\ \end{gathered} \right)$$

For the first DG, rewrite (26) in the matrix form:

29 $${\mathbf{\Delta }}{{\mathbf{\dot {P}}}_{\mathbf{l}}}= - {\omega _c}{\mathbf{\Delta }}{{\mathbf{P}}_l}+{\omega _c}{{\mathbf{K}}_{{\mathbf{Pl}}}}{\mathbf{\Delta \tilde {\delta }}}$$

where$${\mathbf{\Delta \tilde {\delta }}}=[\Delta {\tilde {\delta }_{11}}, \cdots ,\Delta {\tilde {\delta }_{NM}}]$$, and

30 $${{\mathbf{K}}_{{\mathbf{Pl}}}}=\left[ {\begin{array}{*{20}{c}} {k_{{p\_{\delta _{11}}}}^{{11}}}& \cdots &{k_{{p\_{\delta _{NM}}}}^{{11}}} \\ \vdots & \ddots & \vdots \\ {k_{{p\_{\delta _{11}}}}^{{N1}}}& \cdots &{k_{{p\_{\delta _{NM}}}}^{{N1}}} \end{array}} \right]$$

For the other DGs, similarly, (14) is rewritten as:

31 $${\dot {\omega }_{ij}}=\operatorname{sgn} \left( {{Q_{ij}}} \right)m{\dot {P}_{ij}}+k\left( {{\omega ^*} - \frac{1}{M}\sum\limits_{{j=1}}^{M} {{\omega _{ij}}} } \right)$$

32 $$\Delta {\dot {\tilde {\delta }}_{ij}}=\Delta {\omega _{ij}}$$

Equation (31) is reformulated as:

33 $${\mathbf{\Delta }}{{\mathbf{\dot {\omega }}}_{{\mathbf{is}}}}=m{{\mathbf{D}}_{{\mathbf{is}}}}{\mathbf{\Delta }}{{\mathbf{\dot {P}}}_{{\mathbf{is}}}} - \frac{k}{M}{\mathbf{\Delta }}{{\mathbf{\omega }}_{{\mathbf{ia}}}}$$

where$${{\mathbf{D}}_{{\mathbf{is}}}}=diag[\operatorname{sgn} \left( {{Q_{ij}}} \right)]$$ ,$${\mathbf{\Delta }}{{\mathbf{\dot {\omega }}}_{{\mathbf{is}}}}={[\Delta {\dot {\omega }_{i2}}, \cdots ,\Delta {\dot {\omega }_{iM}}]^T}$$, $${\mathbf{\Delta }}{{\mathbf{P}}_{{\mathbf{is}}}}={[\Delta {P_{i2}}, \cdots ,\Delta {P_{iM}}]^T}$$, and

34 $${\mathbf{\Delta }}{{\mathbf{\dot {P}}}_{{\mathbf{is}}}}= - {\omega _c}{\mathbf{\Delta }}{{\mathbf{P}}_{{\mathbf{is}}}}+{\omega _c}{{\mathbf{K}}_{{\mathbf{Pis}}}}{\mathbf{\Delta \tilde {\delta }}}$$

From (33), there is:

35 $${\mathbf{\Delta }}{{\mathbf{\dot {\omega }}}_{\mathbf{s}}}=m{{\mathbf{D}}_{\mathbf{s}}}{\mathbf{\Delta }}{{\mathbf{\dot {P}}}_{\mathbf{s}}} - \frac{k}{M}{\mathbf{\Delta }}{{\mathbf{\omega }}_{{\mathbf{sa}}}}$$

where $${\mathbf{\Delta }}{{\mathbf{\dot {\omega }}}_{\mathbf{s}}}={[{\mathbf{\Delta }}{{\mathbf{\dot {\omega }}}_{{\mathbf{1s}}}}, \cdots ,{\mathbf{\Delta }}{{\mathbf{\dot {\omega }}}_{{\mathbf{Ns}}}}]^T}$$, $${{\mathbf{D}}_{\mathbf{s}}}={[{{\mathbf{D}}_{{\mathbf{1s}}}}, \cdots ,{{\mathbf{D}}_{{\mathbf{Ns}}}}]^T}$$, $${\mathbf{\Delta }}{{\mathbf{P}}_{\mathbf{s}}}={[{\mathbf{\Delta }}{{\mathbf{P}}_{{\mathbf{1s}}}}, \cdots ,{\mathbf{\Delta }}{{\mathbf{P}}_{{\mathbf{Ns}}}}]^T}$$, $${\mathbf{\Delta }}{{\mathbf{\omega }}_{{\mathbf{sa}}}}={[{\mathbf{\Delta }}{{\mathbf{\dot {\omega }}}_{{\mathbf{1a}}}}, \cdots ,{\mathbf{\Delta }}{{\mathbf{\dot {\omega }}}_{{\mathbf{Na}}}}]^T}$$.

Combining (24), (29), (34), (35), the model of the system with the proposed control method is formulated as:

36 $${\mathbf{\dot {X}}}={\mathbf{AX}}$$

where

37 $${\mathbf{X}}={\left[ {{\mathbf{\Delta }}{{\mathbf{P}}_{\mathbf{l}}},{\mathbf{\Delta }}{{\mathbf{P}}_s},{\mathbf{\Delta }}{{\mathbf{\omega }}_{\mathbf{l}}},{\mathbf{\Delta }}{{\mathbf{\omega }}_{\mathbf{s}}},{\mathbf{\Delta \tilde {\delta }}}} \right]^T}$$

38 $${\mathbf{A}}=\left[ {\begin{array}{*{20}{c}} { - {\omega _c}{\mathbf{E}}}&{\mathbf{0}}&{\mathbf{0}}&{\mathbf{0}}&{{\omega _c}{{\mathbf{K}}_{{\mathbf{Pl}}}}} \\ {\mathbf{0}}&{ - {\omega _c}{\mathbf{E}}}&{\mathbf{0}}&{\mathbf{0}}&{{\omega _c}{{\mathbf{K}}_{{\mathbf{Ps}}}}} \\ {{{\mathbf{A}}_{{\mathbf{31}}}}}&{\mathbf{0}}&{{{\mathbf{K}}_{{\mathbf{\omega ll}}}}}&{{{\mathbf{K}}_{{\mathbf{\omega ls}}}}}&{{{\mathbf{A}}_{{\mathbf{35}}}}} \\ {\mathbf{0}}&{ - {\omega _c}m{{\mathbf{D}}_{\mathbf{s}}}}&{{{\mathbf{K}}_{{\mathbf{\omega sl}}}}}&{{{\mathbf{K}}_{{\mathbf{\omega ss}}}}}&{{\omega _c}m{{\mathbf{D}}_{\mathbf{s}}}{{\mathbf{K}}_{{\mathbf{Ps}}}}} \\ {\mathbf{0}}&{\mathbf{0}}&{\mathbf{E}}&{\mathbf{E}}&{\mathbf{0}} \end{array}} \right]$$

39 $${{\mathbf{A}}_{{\mathbf{31}}}}= - m{\omega _c}{{\mathbf{D}}_{\mathbf{l}}}{{\mathbf{H}}_{\mathbf{l}}} - \mu {\omega _c}{{\mathbf{L}}_{\mathbf{l}}}$$

40 $${{\mathbf{A}}_{{\mathbf{35}}}}={\omega _c}m{{\mathbf{D}}_{\mathbf{l}}}{{\mathbf{H}}_{\mathbf{l}}}{{\mathbf{K}}_{{\mathbf{Pl}}}}+\mu {{\mathbf{L}}_{\mathbf{l}}}{{\mathbf{K}}_{{\mathbf{Pl}}}}$$

41 $${{\mathbf{K}}_{{\mathbf{\omega ll}}}}=c{\mathbf{E}},c= - \frac{k}{M}$$

42 $${{\mathbf{K}}_{{\mathbf{\omega sl}}}}=\left[ {\begin{array}{*{20}{c}} c&{}&{} \\ \vdots &{}&{} \\ c&{}&{} \\ {}& \ddots &{} \\ {}&{}&c \\ {}&{}& \vdots \\ {}&{}&c \end{array}} \right]$$

43 $${{\mathbf{K}}_{{\mathbf{\omega l}}s}}=\left[ {\begin{array}{*{20}{c}} c& \cdots &c&{}&{}&{}&{} \\ {}&{}&{}& \ddots &{}&{}&{} \\ {}&{}&{}&{}&c& \cdots &c \end{array}} \right]$$

44 $${{\mathbf{K}}_{{\mathbf{\omega l}}s}}=\left[ {\begin{array}{*{20}{c}} c& \cdots &c&{}&{}&{}&{} \\ \vdots & \ddots & \vdots &{}&{}&{}&{} \\ c& \cdots &c&{}&{}&{}&{} \\ {}&{}&{}& \ddots &{}&{}&{} \\ {}&{}&{}&{}&c& \cdots &c \\ {}&{}&{}&{}& \vdots & \ddots & \vdots \\ {}&{}&{}&{}&c& \cdots &c \end{array}} \right]$$

In (38),${\mathbf{0}}$is the matrix where all elements are equal to 0.${\mathbf{E}}$is a diagonal matrix with ones on the diagonal.

### Eigenvalue

The root locus is depicted based on the parameters shown in Sect. 5 (simulation validations). The system matrix (38) is obtained near the steady state point. The root locus method in this paper is mainly employed to assess the small-signal stability near the steady-state equilibrium point of systems.

**Fig. 6:**
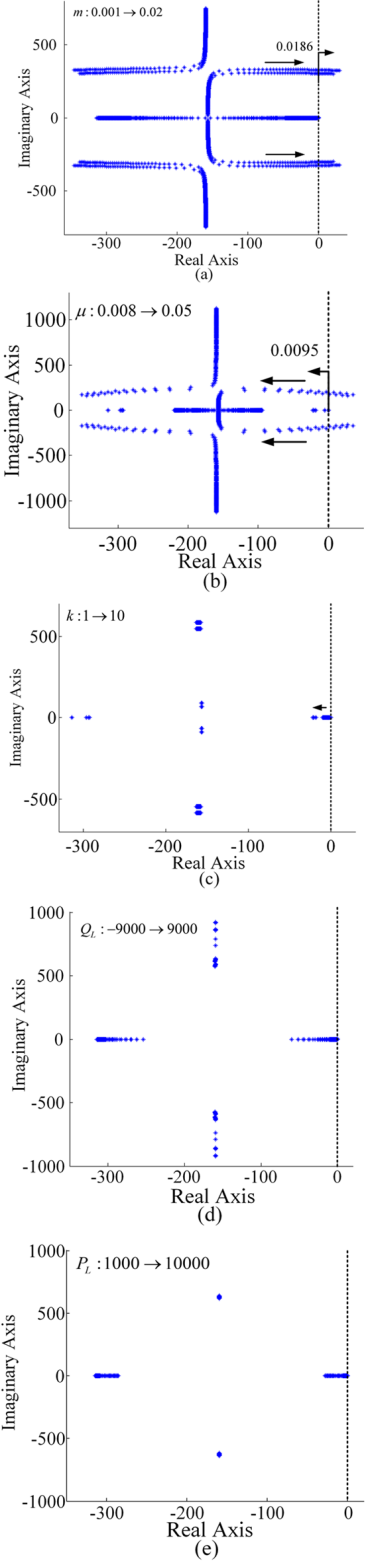
Root locus with parameter variations. **(a)** *m* **(b) **$\mu$** (c)** *k* **(d) **${Q_L}$ **(e) **${P_L}$.

The root locus, with *k*, $\mu$and ${Q_L}$as constants, changes as *m* increases. Setting *k*, $\mu$ and ${Q_L}$ to 5, 0.02, and 9000 respectively, Fig. 6(a) for *m* increasing from 0.001 to 0.0186, all root loci are positioned in the left half-plane^41^. However, when *m* exceeds 0.0186, the eigenvalues move to the right half-plane, resulting in the loss of system stability. Therefore, in this case, the system achieves stability when *m* within the range of [0.001, 0.0186].

The root locus with *m*, *k* and${Q_L}$as constants varies as$\mu$increases. Figure 6(b) illustrates the root locus for$\mu$increasing from 0.008 to 0.05, with *m*, *k* and ${Q_L}$held at 0.01, 5, and 9000, respectively. As$\mu$increases from 0.008 to 0.0095, eigenvalues appear in the right half-plane, rendering the system unstable. However, when$\mu$exceeds 0.0095, eigenvalues move to the left half-plane, and the microgrid remains stable. Therefore, microgrids remain stable when$\mu$is within the range of 0.0095 to 0.05.

The root locus with *m*, $\mu$and ${Q_L}$as constants varies as *k* increases. The root locus diagram for *k* increasing from 1 to 10 is shown in Fig. 6(c), with $m=0.01$, $\mu =0.02$, ${Q_L}=9000$. When *k* increases from 1 to 10, all points stay in the left half-plane. Therefore, the microgrid is stable as $k \in [1,10]$.

The root locus, with control parameters m, $\mu$, *k* as constants, changes as the load varies from RC to RL. To quantify the influence of sign function on stability, the root locus diagram with ${Q_L}$increasing from − 9000 to 9000 is depicted in Fig. 6(d), and $m=0.01$, $\mu =0.02$, $k=5$. Further, the root locus with the active power load disturbances${P_L}$ is displayed in Fig. 6(e), indicating the system is stable. As observed, since all points lie in the left half-plane, the microgrid is stable.

## Simulation validations

**Fig. 7:**
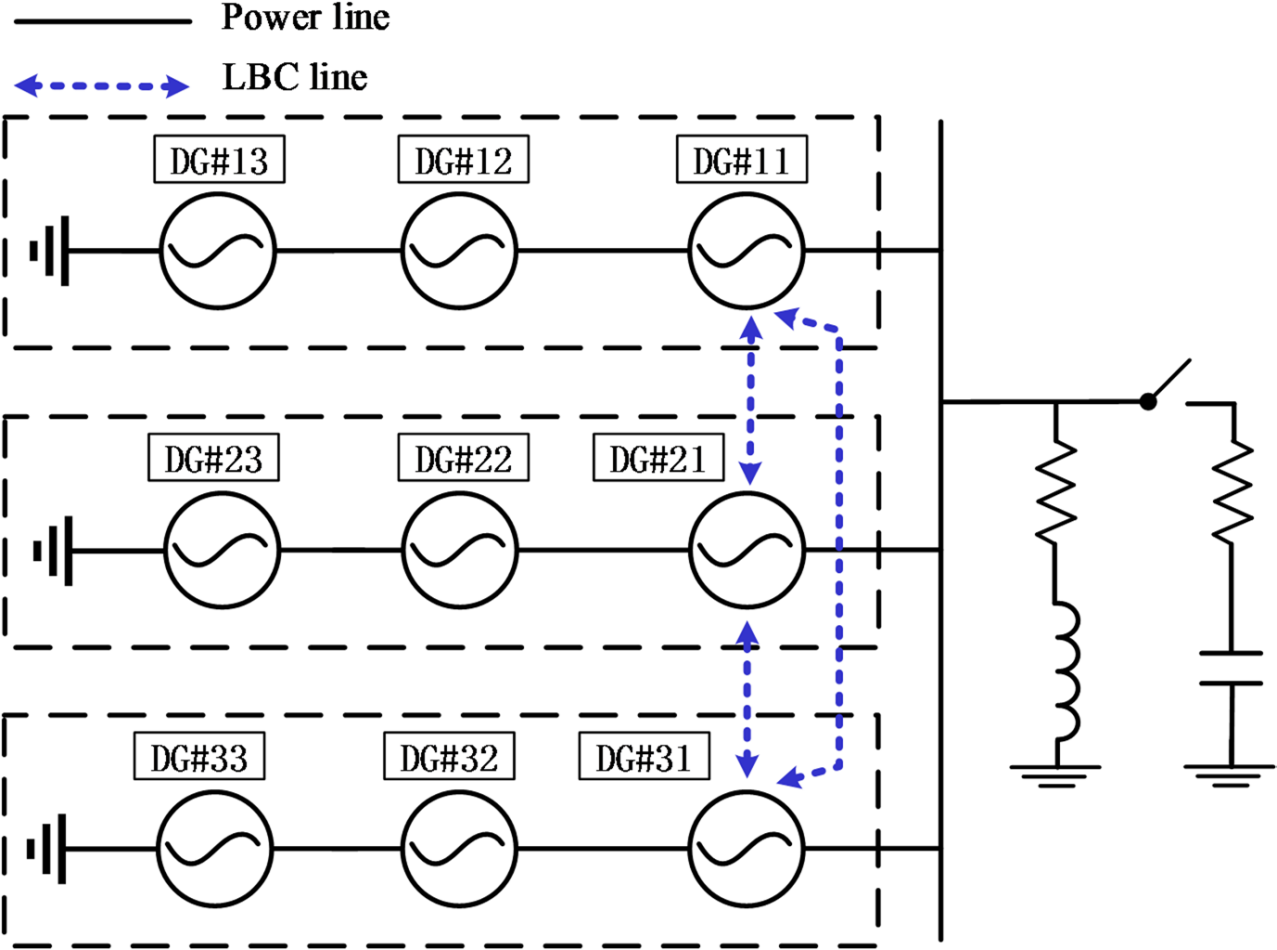
The nine DGs simulation system.

For verifying the effectiveness of the proposed control method, at least 9 DGs (3 × 3) are required. However, due to experimental constraints, this paper has only conducted simulation verifications. The proposed frequency restoration control is validated through simulations conducted on the MATLAB/Simulink platform. The series-parallel-type microgrid comprises 9 DGs (see Fig. 7). Each string contains 3 DGs connected in cascade, then 3 such string modules are connected in parallel. Detailed simulation parameters are provided in Table 2. The switching frequency${f_s}$of DGs is 10 kHz, and the power ratings ${S_{rated}}$for all DGs are 3000VA. The inner loop adopts proportional control (${K_P}$), and the outer loop adopts PI control (${k_P}$,${k_I}$).

**Table 2:** Simulation parameters.

Variables	Values	Variables	Values
$V_{P}^{*}$(V)	311	*m*	0.01
${f^*}$(Hz)	50	$\mu$	0.02
${f_i}$(Hz)	[49, 51]	${L_1}\left( {mH} \right)$	1.01
${L_2}\left( {mH} \right)$	1.02	${L_3}\left( {mH} \right)$	1
*k*	5	${L_f}\left( {mH} \right)$	0.6
${V_{ij}}(V)$	311/3	${C_f}\left( {\mu F} \right)$	20
${f_s}\left( {kHz} \right)$	10	${S_{rated}}\left( {VA} \right)$	3000
${K_P}$	0.3	${k_P}$,${k_I}$	0.1,10

### Case 1: k = 0 without frequency restoration

**Fig. 8:**
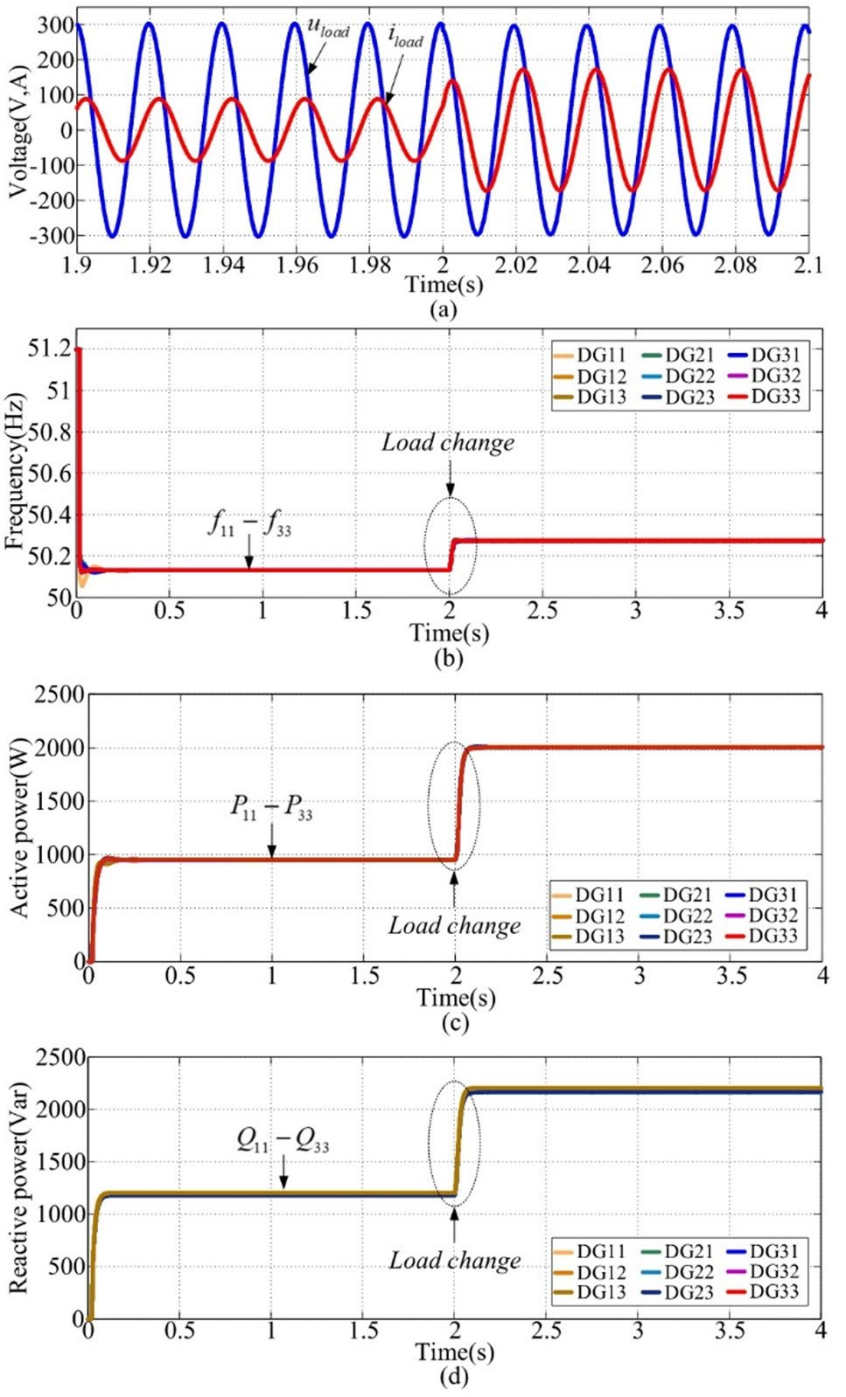
Simulation results with k = 0. **(a)** voltage and current, **(b) **${f_{ij}}$, **(c) **${P_{ij}}$, **(d) **${Q_{ij}}$.

The simulation is conducted to illustrate the proposed method’s performance without frequency restoration (k = 0) under RL load conditions. At t = 2s, a load step occurs. Waveforms of load voltages and currents are shown in Fig. 8(a). It can be observed that load currents increase after t = 2s, and voltage and current waveforms smoothly transition during load changes. The frequency, as shown in Fig. 8(b), increases with the load increase. For RL loads, inverse droop control is used. The frequency of the first interval is 50.12 Hz, and that of the second interval is 50.28 Hz, with errors of 0.12 Hz and 0.28 Hz relative to the nominal value of 50 Hz. Further, the transient response time is about 0.2s in the first interval. The waveform illustrating the equal distribution of active power is shown in Fig. 8(c). Due to impedance mismatch, the reactive power cannot be shared accurately, as shown in Fig. 8(d). Based on these simulation results, the control with k = 0, the frequency will deviate from the nominal value with changes in loads.

### Case 2: k = 5 with frequency restoration

**Fig. 9:**
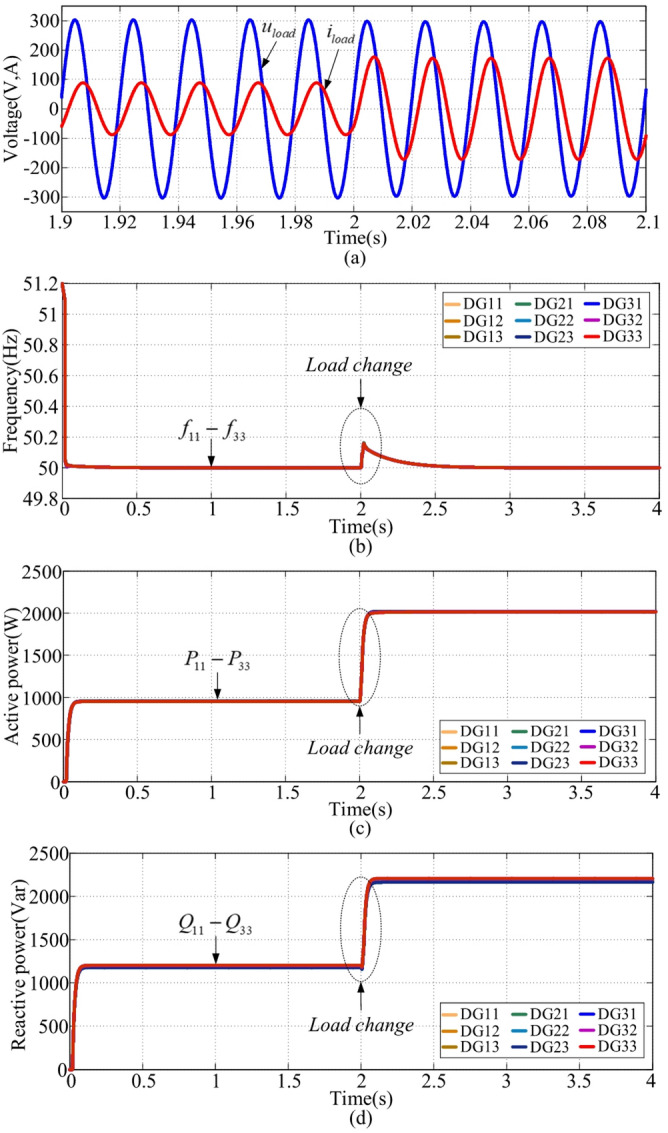
Simulation results with k = 5. **(a)** voltage and current, **(b) **${f_{ij}}$, **(c) **${P_{ij}}$, **(d) **${Q_{ij}}$.

This scenario is performed using the proposed method for RL loads with k = 5 (frequency restoration enabled). With the same load setting as case 1, the load voltage and current waveforms are illustrated in Fig. 9(a), in which they are smooth during the load change. From (11) and (13), the proposed method sets a fixed voltage, which will not drop as the load increases. If neglects the voltage drop along the lines, enabling the load voltage to be controlled within a feasible range. Frequencies, depicted in Fig. 9(b), remain at the nominal value of 50 Hz with the same load setting as case 1. The transient response time is about 0.1s in the first interval. Compared to Fig. 8(b) and Fig. 9(b), the proposed method effectively achieves frequency restoration performance. Active and reactive powers are presented in Fig. 9(c) and (d). Therefore, frequencies could be kept at the nominal value despite load variations.

### Case 3: switching of load types

**Fig. 10:**
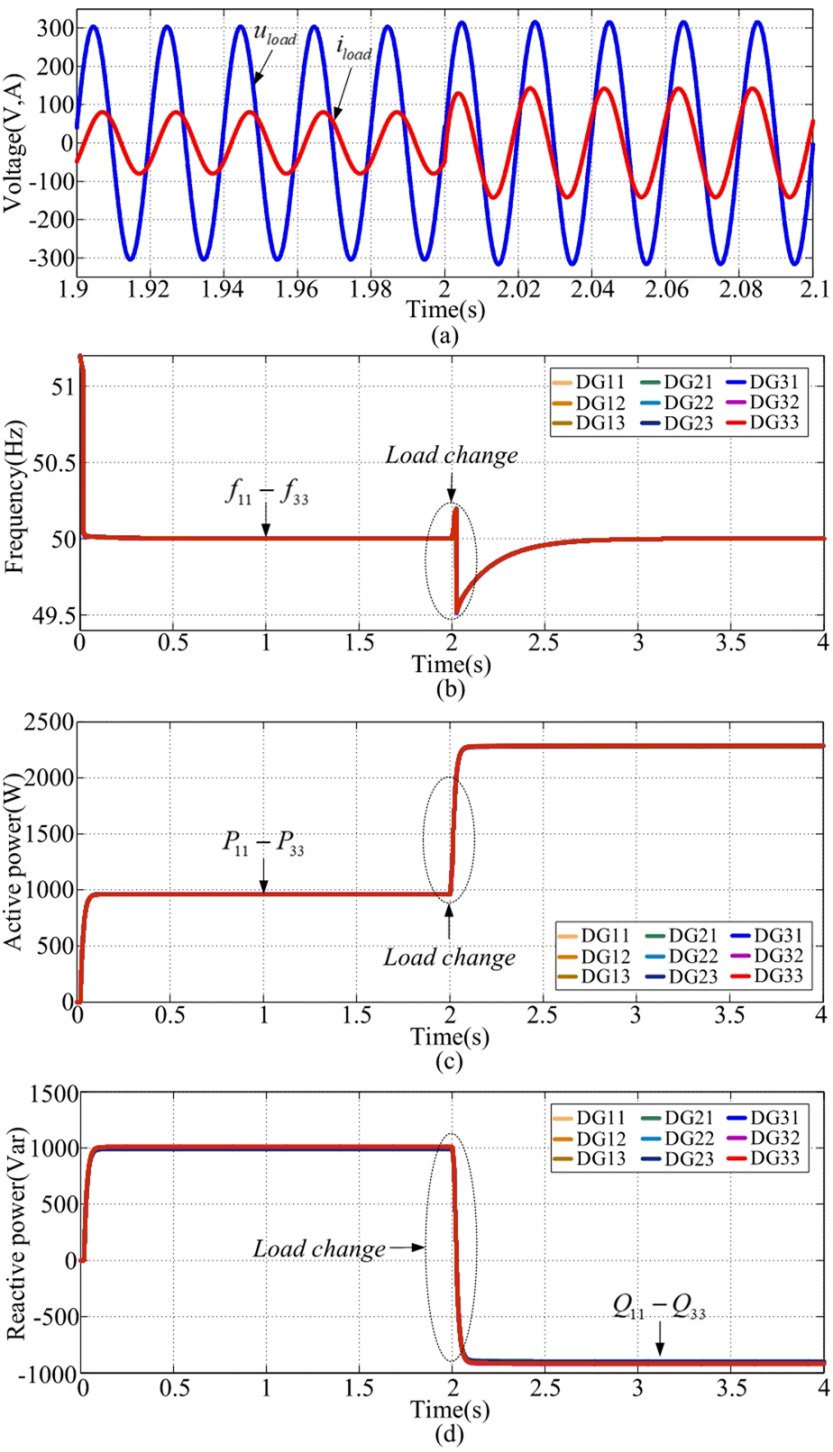
Load switching simulation results. **(a)** voltage and current, **(b) **${f_{ij}}$, **(c) **${P_{ij}}$, **(d) **${Q_{ij}}$.

This scenario involves transitioning from RL to RC loads (X/R load ratios change). Load voltages and currents are displayed in Fig. 10(a). Before t = 2s, the voltage phase angle is ahead of the current phase angle. After t = 2s, the voltage phase angle falls behind the current phase angle. Frequency is presented in Fig. 10(b). With changes in load type, the frequency always remains at the nominal value, in which the deviation error is zero. Active powers and reactive powers are introduced in Fig. 10(c) and (d). Accordingly, the proposed scheme is robust against the switching of load types.

### Case 4: communication failure with the existing secondary centralized frequency restoration control^40^

**Fig. 11:**
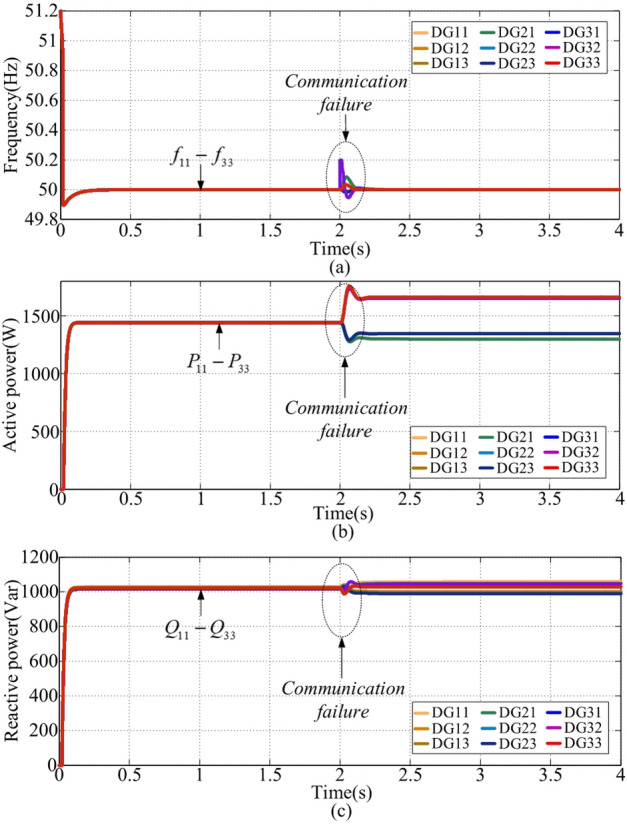
Simulation waveforms of case 4. **(a) **${f_{ij}}$, **(b) **${P_{ij}}$, **(c) **${Q_{ij}}$.

This case is conducted with the secondary centralized frequency restoration control under a single-point communication failure^40^. At t = 2s, a single-point communication failure takes place in DG31. The waveforms for frequency, active power, and reactive power are illustrated in Fig. 11(a), (b), and (c). From Fig. 11(a), after t = 2s, the settling time is about 0.2s, and the overshoot is approximately 0.2 Hz. By analyzing the results before and after t = 2s, it is evident that the secondary centralized frequency restoration control fails to achieve the desired active power-sharing when a single-point communication failure occurs.

### Case 5:communication failure with the proposed control

**Fig. 12:**
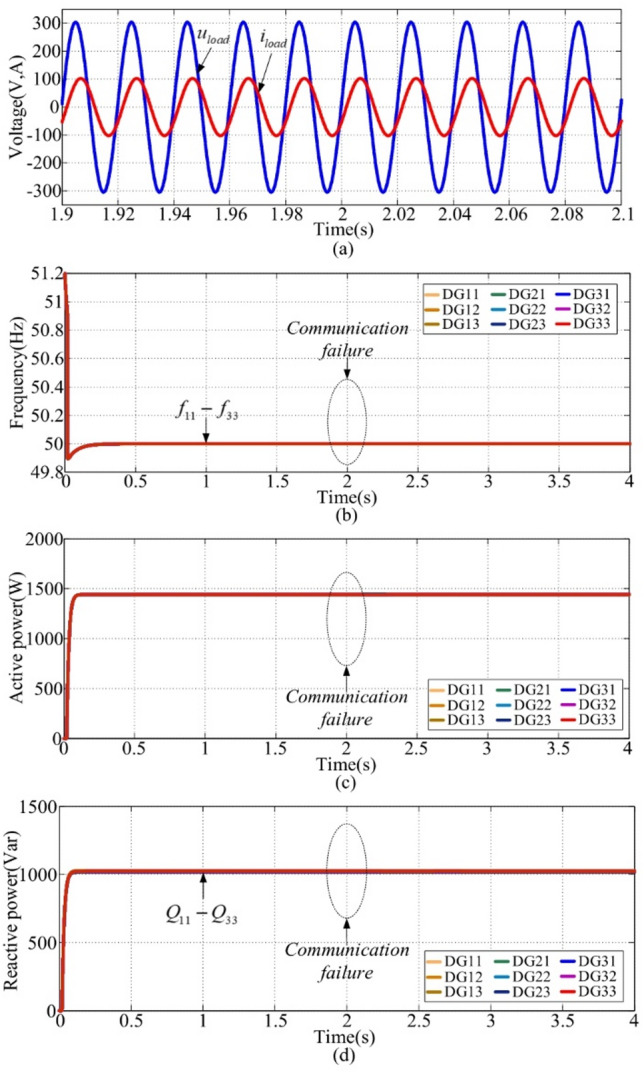
Simulation waveforms of case 5. **(a)** voltage and current, **(b) **${f_{ij}}$, **(c) **${P_{ij}}$, **(d) **${Q_{ij}}$.

This scenario is conducted under communication failure conditions between DG11 and DG31 at t = 2s. The simulation results, including load voltages and currents, frequency, active powers, and reactive powers, are presented in Fig. 12(a), (b), (c), and (d). From Fig. 12(a), after t = 2s, the overshoot and the settling time are zero. The proposed control strategy ensures that the communication failure at t = 2s does not influence the performance of power sharing and frequency restoration. Compared to the results in Figs. 11 and 12, it indicates that the proposed strategy is robust to communication failures to a certain extent, and the reliability of the system is improved.

### Case 6: different power allocations

**Fig. 13:**
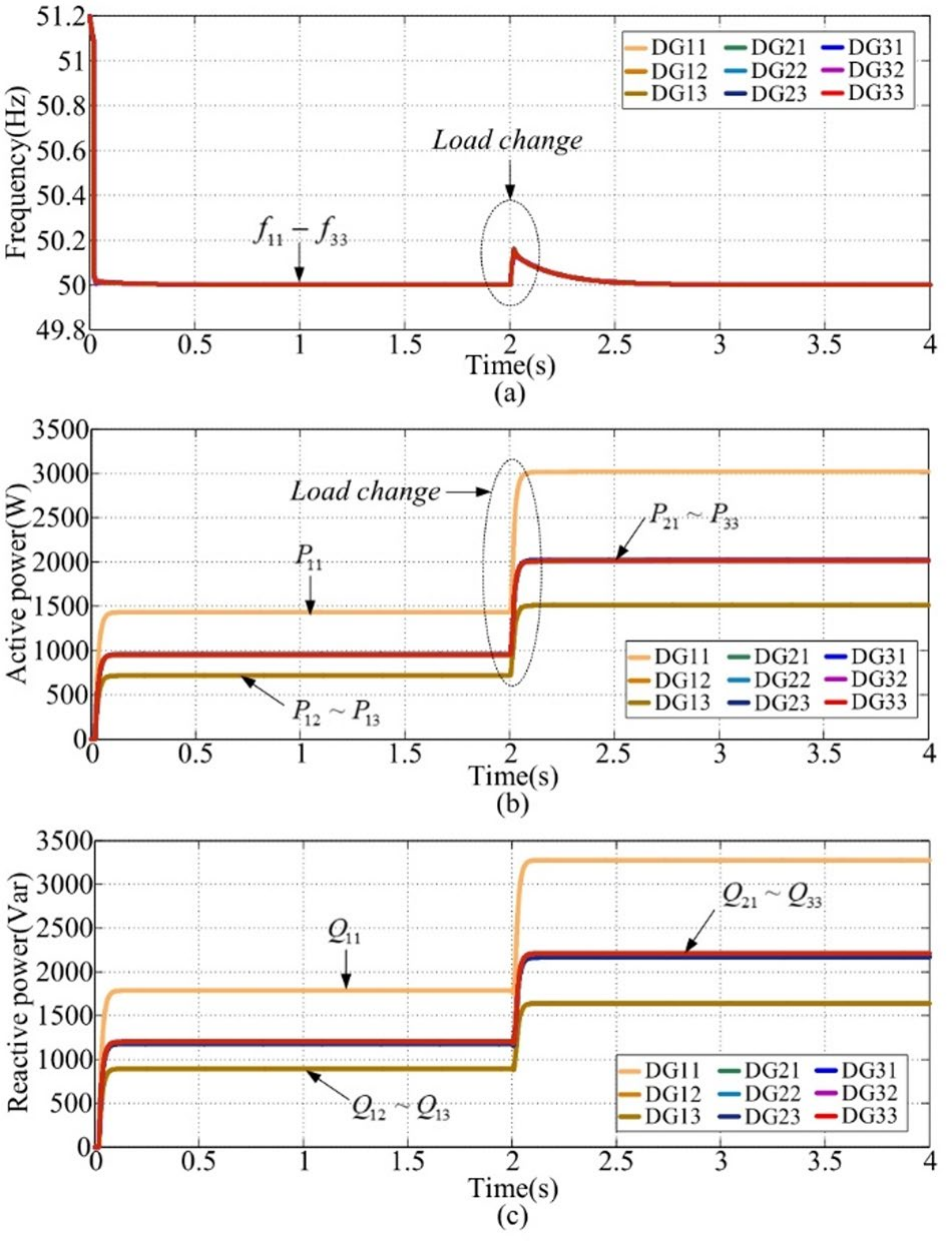
Simulation waveforms of case 6. **(a) **${f_{ij}}$, **(b) **${P_{ij}}$, **(c) **${Q_{ij}}$.

This case verifies the proportional active power-sharing performance of the proposed control strategy. By setting the reference output voltage amplitude of each DG, the active power sharing could be adjusted. In this case, the output voltages of DG11, DG12, and DG13 are set to 311 × (2/4), 311 × (1/4), and 311 × (1/4), respectively, while the voltages of other DGs are set to 311 × (1/3). The frequency waveform, shown in Fig. 13(a), demonstrates that the system’s frequency restoration is maintained despite load variations. The active and reactive power waveforms, shown in Fig. 13(b) and (c), indicate that the active power sharing ratio between DG11 and DG22 is approximately 1:2. Therefore, the proposed control strategy enables proportional power sharing of series-parallel-type microgrids while maintaining frequency restoration.

### Case 7: Open-circuit fault of DG13

**Fig. 14:**
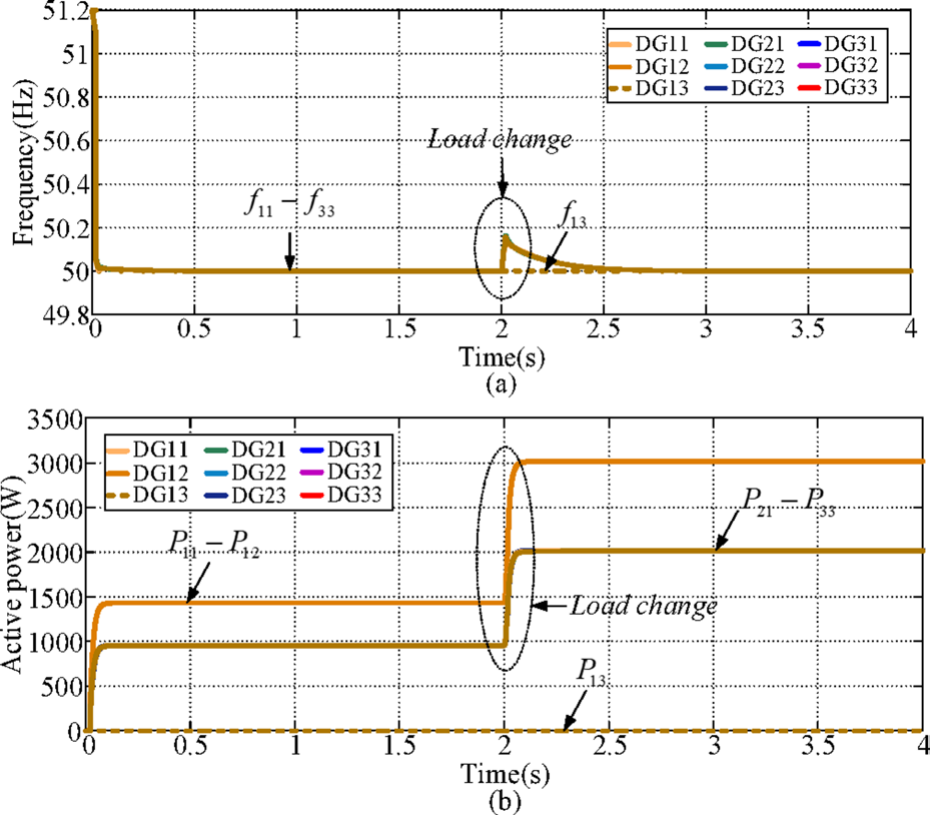
Simulation waveforms of case 7. **(a) **${f_{ij}}$, **(b) **${P_{ij}}$.

In this case, an open-circuit fault is imposed on DG13. A load change is introduced at t = 2 s. The voltage reference values are set to 311/2 V for DG11 and DG12, while the remaining DGs are assigned as 311/3 V. The simulated frequency is shown in Fig. 14 (a). As observed, the proposed controller is still capable of achieving frequency restoration under the DG13 open-circuit condition. The active power waveforms are presented in Fig. 14(b). Since DG13 is disconnected, its output power remains zero throughout the test. Moreover, because the output voltage references of DG11 and DG12 are higher than those of the other DGs, DG11 and DG12 deliver higher active power compared with the remaining units. Therefore, the system maintains effective frequency recovery despite the fault with the proposed controller.

## Conclusion

A frequency restoration control scheme that significantly reduced the communication requirements was proposed for series-parallel-type microgrids. The first DGs in each string were regulated by utilizing only the local LBC. The rest modules in each string were controlled without any communication. The stability analysis and simulation results indicated that the proposed controller achieved frequency restoration, applied to RL and RC loads, maintained robustness under certain communication failures, and ensured proportional active power-sharing performance. Compared to the existing methods, the proposed method has significantly reduced communication requirements and computational complexity, which improved reliability and lowered initial investment costs. This paper proposes a feasible control method for a series-parallel-type microgrid to realize frequency restoration. Similar to the parallel system, the series-parallel-type system holds a potential scalability and plug-and-play capability. However, it remains far from practical engineering implementation, as several real-world challenges have not yet been fully addressed. The limitation of the series-parallel-type microgrid is sensitive to single-point failures.

Based on this research, future work may be extended to series-parallel-type systems with wind PV storage, motor loads, communication delays, faults, renewable intermittency, and higher uncertainty. Further, other series-parallel-type microgrid structures, in which DG strings are connected to different buses and interconnected through power lines, will be studied in future work.

## Data Availability

The data that support the findings of this study are available from the corresponding author upon reasonable request.

## References

[1] Sen, S. & Kumar, V. Simplified modeling and HIL validation of solar PVs and Storage-Based islanded microgrid with generation uncertainties. *IEEE Syst. J.* **14**, 2653–2664 (2020).

[2] Ramesh, M., Yadav, A. K. & Pathak, P. K. A novel fuzzy assisted sliding mode control approach for frequency regulation of wind-supported autonomous microgrid. *Sci. Rep.* **14**, 31526 (2024). 39733153 10.1038/s41598-024-83202-z PMC11682459

[3] Şen, M., Özcan, M. & Eker, Y. R. Fuzzy Logic-Based energy management system for regenerative braking of electric vehicles with hybrid energy storage system. *Appl. Sci.* **14**, 3077 (2024).

[4] Samimi, A., Nikzad, M. & Zakipour, A. Optimal sizing model of battery energy storage in a droop-controlled islanded multi-carrier microgrid based on an advanced frequency droop model. *Sci. Rep.* **15**, 2540 (2025). 39833259 10.1038/s41598-025-86368-2 PMC11747571

[5] Louassaa, K., Guerrero, J. M. & Boukerdja, M. A novel hierarchical control strategy for enhancing stability of a DC microgrid feeding a constant power load. *Sci. Rep.* **15**, 7061 (2025). 40016478 10.1038/s41598-025-89318-0 PMC11868593

[6] Habibi, S. I. et al. Multiagent-Based nonlinear generalized minimum variance control for islanded AC microgrids. *IEEE Trans. Power Syst.* **39**, 316–328 (2024).

[7] Baharizadeh, M. & Karshenas, H. R. A decentralized control for accurate power sharing and precise voltage regulation in hybrid Single-Phase AC/DC microgrids. *IEEE Trans. Smart Grid*. **15**, 2493–2506 (2024).

[8] Sarrafan, N. et al. Improved distributed prescribed Finite-Time secondary control of Inverter-Based microgrids: design and Real-Time implementation. *IEEE Trans. Industr. Electron.* **68**, 11135–11145 (2021).

[9] Mohammed, N., Udawatte, H., Zhou, W., Hill, D. J. & Bahrani, B. Grid-Forming inverters: A comparative study of different control strategies in frequency and time domains. *IEEE Open. J. Industrial Electron. Soc.* **5**, 185–214 (2024).

[10] Kikusato, H. et al. Performance evaluation of grid-following and grid-forming inverters on frequency stability in low-inertia power systems by power hardware-in-the-loop testing. *Energy Rep.* **9**, 381–392 (2023).

[11] Bahrani, B. Power-Synchronized Grid-Following inverter without a Phase-Locked loop. *IEEE Access.* **9**, 112163–112176 (2021).

[12] Teng, Y. et al. Review on grid-forming converter control methods in high-proportion renewable energy power systems. *Global Energy Interconnect.* **5**, 328–342 (2022).

[13] Chakraborty, S., Patel, S. & Salapaka, M. µ-Synthesis-Based generalized robust framework for Grid-Following and Grid-Forming inverters. *IEEE Trans. Power Electron.* **38**, 3163–3179 (2023).

[14] Liu, J. et al. Decentralized secondary frequency control of autonomous microgrids via adaptive Robust-Gain performance. *IEEE Trans. Smart Grid*. **15**, 67–76 (2024).

[15] Shan, Y., Hu, J. & Shen, B. Distributed secondary frequency control for AC microgrids using load power forecasting based on artificial neural network. *IEEE Trans. Industr. Inf.* **20**, 1651–1662 (2024).

[16] Guo, F. et al. Decentralized cluster-Based distributed secondary control of Large-Scale DC microgrid cluster system. *IEEE Trans. Sustain. Energy*. **15**, 1652–1662 (2024).

[17] Lian, Z., Wen, C., Guo, F., Lin, P. & Wu, Q. Decentralized secondary control for frequency restoration and power allocation in islanded AC microgrids. *Int. J. Electr. Power Energy Syst.* **148**, 108927 (2023).

[18] Shi, Y., Liu, Z., Wang, J., Liu, J. A. & Small -AC-Signal injection based decentralized secondary voltage control for parallel inverters with accurate reactive power sharing in islanded microgrids. *IEEE Trans. Power Electron.* **38**, 14573–14589 (2023).

[19] Liberos, M. et al. A control stage for Parallel-Connected interlinking converters in hybrid AC–DC microgrids. *IEEE Access.* **11**, 61800–61812 (2023).

[20] Liu, Y., Li, Z. & Zhao, J. Safety-Constrained stagewise optimization of droop control parameters for isolated microgrids. *IEEE Trans. Smart Grid*. **15**, 77–88 (2024).

[21] Karmakar, B. K. & Pradhan, A. K. Performance Analysis of P-V And Q-F Droop Control Strategy In An Islanded Resistive Microgrid During Partial Shading On Photovoltaic Plant. 2018 International Conference on Recent Innovations in Electrical, Electronics & Communication Engineering (ICRIEECE), Bhubaneswar, India, 1289–1294 (2018).

[22] Li, Z. et al. Distributed Event-Triggered secondary control for economic dispatch and frequency restoration control of Droop-Controlled AC microgrids. *IEEE Trans. Sustain. Energy*. **11**, 1938–1950 (2020).

[23] Gómez, N. F. A., Llanos, J. S., Rute, J., Sáez, E. & Sumner, D. Distributed predictive control strategy for frequency restoration of microgrids considering optimal dispatch. *IEEE Trans. Smart Grid*. **12**, 2748–2759 (2021).

[24] Rey, J. M. et al. Local frequency restoration for Droop-Controlled parallel inverters in islanded microgrids. *IEEE Trans. Energy Convers.* **34**, 1232–1241 (2019).

[25] Ullah, S., Khan, L., Sami, I. & Ullah, N. Consensus-Based Delay-Tolerant distributed secondary control strategy for droop controlled AC microgrids. *IEEE Access.* **9**, 6033–6049 (2021).

[26] Ullah, S., Khan, L., Sami, I. & Ro, J. Voltage/Frequency regulation with optimal load dispatch in microgrids using SMC based distributed cooperative control. *IEEE Access.* **10**, 64873–64889 (2022).

[27] Ullah, S. et al. *A Finite-Time Robust Distributed Cooperative Secondary Control Protocol for Droop-Based Islanded AC Microgrids* Vol. 14, 2936 (Energies, 2021).

[28] Das, S., Nutkani, I. U. & Teixeira, C. A. Decentralized Master-Slave control for Series-Cascaded islanded AC microgrid. *IEEE Trans. Industr. Electron.* **69**, 5942–5951 (2022).

[29] Xiao, Q. et al. Review of fault diagnosis and fault-Tolerant control methods of the modular multilevel converter under submodule failure. *IEEE Trans. Power Electron.* **38**, 12059–12077 (2023).

[30] He, J., Li, Y., Liang, B. & Wang, C. Inverse power factor droop control for decentralized power sharing in Series-Connected-Microconverters-Based islanding microgrids. *IEEE Trans. Industr. Electron.* **64**, 7444–7454 (2017).

[31] Li, L. et al. Power factor angle consistency control for decentralized power sharing in Cascaded-type microgrid. *IET Generation Transmission Distribution*. **13**, 850–857 (2019).

[32] Li, L., Sun, Y., Liu, Z., Su, M. A. & Communication-Free Economic dispatch control scheme of Cascaded-Type microgrids for operating cost minimization. *IEEE J. Emerg. Sel. Top. Power Electron.* **12**, 1974–1983 (2024).

[33] Qin, C., Li, X. A., Novel Distributed & Hierarchical Control Method for the Series-Parallel-Type Microgrid. and. 2023 China Automation Congress (CAC), Chongqing, China, 6770–6775 (2023).

[34] He, J. et al. Hybrid microgrid with Parallel- and Series-Connected microconverters. *IEEE Trans. Power Electron.* **33**, 4817–4831 (2018).

[35] Han, H., Zhu, Y., Shi, G., Su, M. & Sun, Y. A. Local-Distributed and Global-Decentralized SoC balancing method for hybrid Series-Parallel energy storage system. *IEEE Syst. J.* **16**, 2289–2299 (2022).

[36] Li, L., Sun, Y., Hou, X., Zhou, K. & Su, M. A minimal-communication control scheme of series-parallel microgrids for power sharing and frequency synchronization. *Int. J. Electr. Power Energy Syst.* **162**, 110218 (2024).

[37] Yuan, W., Wang, Y., Ge, X., Hou, X. & Han, H. A. Unified distributed control strategy for hybrid Cascaded-Parallel microgrid. *IEEE Trans. Energy Convers.* **34**, 2029–2040 (2019).

[38] Han, H. et al. Review of power sharing control strategies for islanding operation of AC microgrids. *IEEE Trans. Smart Grid*. **7**, 200–215 (2016).

[39] FU, S. et al. General [P Q]-[ω V] model of hybrid GFM/GFL Multi-VSC systems: power Oscillation analysis and suppression method. *IEEE J. Emerg. Sel. Top. Industrial Electron.* **5**, 1362–1375 (2024).

[40] Ahmed, K., Seyedmahmoudian, M., Mekhilef, S., Mubarak, N. M. & Stojcevski, A. A. Review on primary and secondary controls of Inverter-interfaced microgrid. *J. Mod. Power Syst. Clean. Energy*. **9**, 969–985 (2021).

[41] Simpson-Porco, J. W., Florian, D. & Francesco, B. Synchronization and power sharing for Droop-Controlled inverters in islanded microgrids. *Automatica* **49**, 2603–2611 (2013).

